# Cognitive biomarker prioritization in Alzheimer’s Disease using brain morphometric data

**DOI:** 10.1186/s12911-020-01339-z

**Published:** 2020-12-02

**Authors:** Bo Peng, Xiaohui Yao, Shannon L. Risacher, Andrew J. Saykin, Li Shen, Xia Ning, for the ADNI

**Affiliations:** 1 The Ohio State University, Columbus, USA; 2 University of Pennsylvania, Philadelphia, USA; 3 Indiana University, Indianapolis, USA

**Keywords:** Alzheimer’s Disease, Learning to rank, Bioinformatics, Machine learning

## Abstract

**Background:**

Cognitive assessments represent the most common clinical routine for the diagnosis of Alzheimer’s Disease (AD). Given a large number of cognitive assessment tools and time-limited office visits, it is important to determine a proper set of cognitive tests for different subjects. Most current studies create guidelines of cognitive test selection for a targeted population, but they are not customized for each individual subject. In this manuscript, we develop a machine learning paradigm enabling personalized cognitive assessments prioritization.

**Method:**

We adapt a newly developed learning-to-rank approach ${\mathtt {PLTR}}$ to implement our paradigm. This method learns the latent scoring function that pushes the most effective cognitive assessments onto the top of the prioritization list. We also extend ${\mathtt {PLTR}}$ to better separate the most effective cognitive assessments and the less effective ones.

**Results:**

Our empirical study on the ADNI data shows that the proposed paradigm outperforms the state-of-the-art baselines on identifying and prioritizing individual-specific cognitive biomarkers. We conduct experiments in cross validation and level-out validation settings. In the two settings, our paradigm significantly outperforms the best baselines with improvement as much as 22.1% and 19.7%, respectively, on prioritizing cognitive features.

**Conclusions:**

The proposed paradigm achieves superior performance on prioritizing cognitive biomarkers. The cognitive biomarkers prioritized on top have great potentials to facilitate personalized diagnosis, disease subtyping, and ultimately precision medicine in AD.

## Background

Identifying structural brain changes related to cognitive impairments is an important research topic in Alzheimer’s Disease (AD) study. Regression models have been extensively studied to predict cognitive outcomes using morphometric measures that are extracted from structural magnetic resonance imaging (MRI) scans [1, 2]. These studies are able to advance our understanding on the neuroanatomical basis of cognitive impairments. However, they are not designed to have direct impacts on clinical practice. To bridge this gap, in this manuscript we develop a novel learning paradigm to rank cognitive assessments based on their relevance to AD using brain MRI data.

Cognitive assessments represent the most common clinical routine for AD diagnosis. Given a large number of cognitive assessment tools and time-limited office visits, it is important to determine a proper set of cognitive tests for the subjects. Most current studies create guidelines of cognitive test selection for a targeted population [3, 4], but they are not customized for each individual subject. In this work, we develop a novel learning paradigm that incorporate the ideas of precision medicine and customizes the cognitive test selection process to the characteristics of each individual patient. Specifically, we conduct a novel application of a newly developed learning-to-rank approach, denoted as ${\mathtt {PLTR}}$ [5], to the structural MRI and cognitive assessment data of the Alzheimer’s Disease Neuroimaging Initiative (ADNI) cohort [6]. Using structural MRI measures as the individual characteristics, we are able to not only identify individual-specific cognitive biomarkers but also prioritize them and their corresponding assessment tasks according to AD-specific abnormality. We also extend ${\mathtt {PLTR}}$ to ${\mathtt {PLTR_h}}$ using hinge loss [7] to more effectively prioritize individual-specific cognitive biomarkers. The study presented in this manuscript is a substantial extension from our preliminary study [8].

Our study is unique and innovative from the following two perspectives. First, conventional regression-based studies for cognitive performance prediction using MRI data focus on identifying relevant imaging biomarkers at the population level. However, our proposed model aims to identify AD-relevant cognitive biomarkers customized to each individual patient. Second, the identified cognitive biomarkers and assessments are prioritized based on the individual’s brain characteristics. Therefore, they can be used to guide the selection of cognitive assessments in a personalized manner in clinical practice; it has the potential to enable personalized diagnosis and disease subtyping.

### Literature review

#### Learning to rank

Learning-to-Rank (${\mathtt {LETOR}}$) [9] is a popular technique used in information retrieval [10], web search [11] and recommender systems [12]. Existing ${\mathtt {LETOR}}$ methods can be classified into three categories [9]. The first category is point-wise methods [13], in which a function is learned to score individual instance, and then instances are sorted/ranked based on their scores. The second category is pair-wise methods [14], which maximize the number of correctly ordered pairs in order to learn the optimal ranking structure among instances. The last category is list-wise methods [15], in which a ranking function is learned to explicitly model the entire ranking. Generally, pairwise and listwise methods have superior performance over point-wise methods due to their ability to leverage order structure among instances in learning [9]. Recently, ${\mathtt {LETOR}}$ has also been applied in drug discovery and drug selection [16–19]. For example, Agarwal et al. [20] developed a bipartite ranking method to prioritize drug-like compounds. He et al. [5] developed a joint push and learning-to-rank method to select cancer drugs for each individual patient. These studies demonstrate the great potential of ${\mathtt {LETOR}}$ in computational biology and computational medicine, particularly for biomarker prioritization.

#### Machine learning for AD biomarker discovery

The importance of using big data to enhance AD biomarker study has been widely recognized [6]. As a result, numerous data-driven machine learning models have been developed for early AD detection and AD-relevant biomarker identification including cognitive measures. These models are often designed to accomplish tasks such as classification (e.g., [21]), regression (e.g., [1, 2, 22]) or both (e.g., [23, 24]), where imaging and other biomarker data are used to predict diagnostic, cognitive and/or other outcome(s) of interest. A drawback of these methods is that, although outcome-relevant biomarkers can be identified, they are identified at the population level and not specific to any individual subject. To bridge this gap, we adapt the ${\mathtt {PLTR}}$ method for biomarker prioritization at the individual level, which has greater potential to directly impact personalized diagnosis.

## Methods

### Materials

The imaging and cognitive data used in our study were obtained from the Alzheimer’s Disease Neuroimaging Initiative (ADNI) database [6]. The ADNI was launched in 2003 as a public-private partnership, led by Principal Investigator Michael W. Weiner, MD. The primary goal of ADNI has been to test whether serial MRI, PET, other biological markers, and clinical and neuropsychological assessment can be combined to measure the progression of mild cognitive impairment (MCI, a prodromal stage of AD) and early AD. For up-to-date information, Please refer to [25] for more detailed, up-to-date information.

Participants include 819 ADNI-1 subjects with 229 healthy control (HC), 397 MCI and 193 AD participants. We consider both MCI and AD subjects as patients, and thus we have 590 cases and 229 controls. We downloaded the 1.5T baseline MRI scans and cognitive assessment data from the ADNI website [25]. We processed the MRI scans using Freesurfer version 5.1 [26], where volumetric and cortical thickness measures of 101 regions relevant to AD were extracted to characterize brain morphometry.

We focus our analysis on 151 scores assessed in 15 neuropsychological tests. For convenience, we denote these measures as *cognitive features* and these tests as *cognitive tasks*. The 15 studied tasks include Alzheimer’s Disease Assessment Scale (ADAS), Clinical Dementia Rating Scale (CDR), Functional Assessment Questionnaire (FAQ), Geriatric Depression Scale (GDS), Mini-Mental State Exam (MMSE), Modified Hachinski Scale (MODHACH), Neuropsychiatric Inventory Questionnaire (NPIQ), Boston Naming Test (BNT), Clock Drawing Test (CDT), Digit Span Test (DSPAN), Digit Symbol Test (DSYM), Category Fluency Test (FLUENCY), Weschler’s Logical Memory Scale (LOGMEM), Rey Auditory Verbal Learning Test (RAVLT) and Trail Making Test (TRAIL).

### Joint push and learning-to-rank using scores—${\mathtt {PLTR}}$

We use the joint push and learning-to-rank method that we developed in He et al. [5], denoted as ${\mathtt {PLTR}}$, for personalized cognitive feature prioritization. ${\mathtt {PLTR}}$ has also been successfully applied in our preliminary study [8]. We aim to prioritize cognitive features for each individual patient that are most relevant to his/her disease diagnosis. We will use patients’ brain morphometric measures that are extracted from their MRI scans for the cognitive feature prioritization. The cognitive features are in the form of scores or answers in the cognitive tasks that the patients take. The prioritization outcomes can potentially be used in clinical practice to suggest the most relevant cognitive features or tasks that can most effectively facilitate diagnosis of an individual subject.

In order to prioritize MCI/AD cognitive features, ${\mathtt {PLTR}}$ learns and uses patient latent vector representations and their imaging features to score each cognitive feature for each individual patient. Then, ${\mathtt {PLTR}}$ ranks the cognitive features based on their scores. Patients with similar imaging feature profiles will have similar latent vectors and thus similiar ranking of cognitive features [27, 28]. During the learning, ${\mathtt {PLTR}}$ explicitly pushes the most relevant cognitive features on top of the less relevant features for each patient, and therefore optimizes the latent patient vectors and cognitive feature vectors in a way that they will reproduce the feature ranking structures [9]. In ${\mathtt {PLTR}}$, these latent vectors are learned via solving the following optimization problem:

1 $$\begin{aligned} \min _{U, V} {{\mathcal {L}}_s} = (1 - \alpha ) {P^{\uparrow }_s} + \alpha {O^{ {+}}_s} + \frac{\beta }{2} R_{uv} + \frac{\gamma }{2} R_{\text {csim}}, \end{aligned}$$

where $\alpha$, $\beta$ and $\gamma \in [0,1]$ are coefficients of $O^{{+}}_s$, $R_{uv}$ and $R_{\text {csim}}$ terms, respectively; $U = [{\mathbf {u}}_1, {\mathbf {u}}_2, \cdots , {\mathbf {u}}_m]$ and $V=[{\mathbf {v}}_1, {\mathbf {v}}_2, \cdots , {\mathbf {v}}_n]$ are the latent matrices for patients and features, respectively (${\mathbf {u}}$ and ${\mathbf {v}}$ are column latent patient vector and feature vector, respectively); ${{\mathcal {L}}_s}$ is the overall loss function. In Problem 1, ${P^{\uparrow }_s}$ measures the average number of relevant cognitive features ranked below an irrelevant cognitive feature, defined as follows,

2 $$\begin{aligned} {P^{\uparrow }_s} = \sum \limits _{p = 1}^m \frac{1}{n_p^{ {+}}n_p^{ {-}}}\sum \limits _{{f ^{ {-}}_i\in {{\mathcal {P}}} _p}} \sum \limits _{{f _j^{ {+}}\in {{\mathcal {P}}} _p^{ {+}}}} {\mathbb {I}} ( {s_p}(f _j^{ {+}}) \le {s_p}(f _i^{ {-}}) ), \end{aligned}$$

where *m* is the number of patients, $f ^{ {+}}_j$ and $f ^{ {-}}_i$ are the relevant and irrelevant features of patient ${{\mathcal {P}}} _p$, $n^{ {+}}_p$ and $n^{ {-}}_p$ are their respective numbers, and ${\mathbb {I}}(x)$ is the indicator function (${\mathbb {I}}(x) = 1$ if *x* is true, otherwise 0). In Eq. (2), $s_p(f _i)$ is a scoring function defined as follows,

3 $$\begin{aligned} s_p(f _i) = {\mathbf {u}}^{{\mathsf {T}}}_p {\mathbf {v}}_i, \end{aligned}$$

that is, it calculates the score of feature $f _i$ on patient ${{\mathcal {P}}} _p$ using their respective latent vectors ${\mathbf {u}}_p$ and ${\mathbf {v}}_i$ [29]. By minimizing $P^{\uparrow }_s$, ${\mathtt {PLTR}}$ learns to assign higher scores to relevant features than irrelevant features so as to rank the relevant features at the top of the final ranking list. Note that, ${\mathtt {PLTR}}$ learns different latent vectors and ranking lists for different subjects, and therefore enables personalized feature prioritization. In Problem (1), ${O^{{+}}_s}$ measures the ratio of mis-ordered feature pairs over the relevant features among all the subjects, defined as follows,

4 $$\begin{aligned} \begin{aligned} {O^{ {+}}_s} = \sum \limits _{p=1}^m \frac{1}{|\{f _i^{ {+}} \succ _{{{{\mathcal {P}}} _p}} f _j^{ {+}}\}|} \sum \limits _{{f _i^{ {+}} \succ _{{{{\mathcal {P}}} _p}} f _j^{ {+}}}} {\mathbb {I}}({s_p}(f _i^{ {+}}) < {s_p}(f _j^{ {+}})), \end{aligned} \end{aligned}$$

where $f _i \succ _{{{{\mathcal {P}}} _p}} f _j$ represents that $f _i$ is ranked higher than $f _j$ for patient ${{\mathcal {P}}} _p$. By minimizing $O^{\uparrow }_s$, ${\mathtt {PLTR}}$ learns to push the most relevant features on top of the less relevant features. Thus, most relevant features are pushed to the very top of the ranking list. In Problem (1), $R_{uv}$ is a regularizer on *U* and *V* to prevent overfitting, defined as,

5 $$\begin{aligned} R_{uv} = \frac{1}{m}\Vert U\Vert ^2_{{F}} + \frac{1}{n}\Vert V\Vert ^2_{{F}}, \end{aligned}$$

where $\Vert X\Vert _{{F}}$ is the Frobenius norm of matrix *X*. $R_{\text {csim}}$ is a regularizer on patients to constrain patient latent vectors, defined as

6 $$\begin{aligned} R_{\text {csim}} = \frac{1}{m^2}\sum _{p=1}^m\sum _{q=1}^m w_{pq}\Vert {\mathbf {u}}_p-{\mathbf {u}}_q\Vert _2^2, \end{aligned}$$

where $w_{pq}$ is the similarity between subject ${{\mathcal {P}}} _p$ and ${{\mathcal {P}}} _q$ that is calculated using the imaging features of the subjects. The assumption here is that patients who are similar in terms of imaging features could also be similar in terms of cognitive features.

### Joint push and learning-to-rank with marginalization—${\mathtt {PLTR_h}}$

The objective of ${\mathtt {PLTR}}$ is to score relevant features higher than less relevant features as shown in Eqs. 2 and 4. However, in some cases, the score of relevant features is expected to be higher than that of less relevant features by a large margin. For example, patients can be very sensitive to a few cognitive tasks but less sensitive to many others. In order to incorporate such information, we propose a new hinge loss [7] based ${\mathtt {PLTR}}$, denoted as ${\mathtt {PLTR_h}}$. In ${\mathtt {PLTR_h}}$, the overall loss function is very similar to Eq. 1, defined as follows,

7 $$\begin{aligned} \min _{U, V} {{\mathcal {L}}_h} = (1 - \alpha ) {P^{\uparrow }_h} + \alpha {O^{ {+}}_h} + \frac{\beta }{2} R_{uv} + \frac{\gamma }{2} R_{\text {csim}}, \end{aligned}$$

where ${{\mathcal {L}}_h}$ is the overall loss function; *U*, *V*, $R_{uv}$ and $R_{\text {csim}}$ are identical as those in Eq. 1. In ${\mathtt {PLTR_h}}$, ${P^{\uparrow }_h}$ measures the average loss between the relevant features and irrelevant features using hinge loss as follows,

8 $$\begin{aligned} \begin{aligned} {P^{\uparrow }_h} = \sum \limits _{{p = 1}}^m \frac{1}{n_p^{ {+}}n_p^{ {-}}}\!\!\sum \limits _{{{f ^{ {-}}_i\in {{\mathcal {P}}} _p}}} \sum \limits _{{{~~f _j^{ {+}}\in {{\mathcal {P}}} _p^{ {+}}}}} {\text{max}} (0, t_p\, -\, ( {s_p}(f _j^{ {+}})\, -\, {s_p}(f _i^{ {-}}) ) ), \end{aligned} \end{aligned}$$

where ${\text {max}} (0, t_p - ( {s_p}(f _j^{ {+}}) - {s_p}(f _i^{ {-}}) ) )$ is the hinge loss (${\text {max}}(0, x) = x {\text { if }}x>0$, otherwise 0) between the relevant feature $f _j^{ {+}}$ and the irrelevant feature $f _i^{ {-}}$, and $t_p$ is the pre-defined margin. Specifically, only when ${s_p}(f _j^{ {+}}) - {s_p}(f _i^{ {-}}) > t_p$ will not induce any loss during optimization. Otherwise, the hinge loss will be positive and increase as ${s_p}(f _j^{ {+}}) - {s_p}(f _i^{ {-}})$ gets smaller than $t_p$. Thus, the hinge loss forces the scores of relevant features higher than those of irrelevant features by at least $t_p$. By doing this, the relevant features are ranked higher than irrelevant features in the ranking list. Similarly, ${O^{ {+}}_h}$ measures the average loss among the relevant features also using hinge loss as follows,

9 $$\begin{aligned} \begin{aligned} {O^{ {+}}_h} = \sum \limits _{p=1}^m \frac{1}{|\{f _i^{ {+}}\!\!\succ _{{{{{\mathcal {P}}} _p}}} f _j^{ {+}}\}|} \sum \limits _{{~~~{f _i^{ {+}} \succ _{{{{\mathcal {P}}} _p}} f _j^{ {+}}}}} {\text {max}} (0, t_o - ( {s_p}(f _i^{ {+}}) - {s_p}(f _j^{ {-}}) ) ), \end{aligned} \end{aligned}$$

where $t_o$ is also the pre-defined margin.

### Data processing

#### Data normalization

Following the protocol in our preliminary study [8], we selected all the MCI and AD patients from ADNI and conducted the following data normalization for these patients. We first performed a *t* test on each cognitive feature between patients and controls, and selected those features if there is a significant difference between patients and controls on these features. Then, we converted the selected features into [0, 1] by shifting and scaling the feature values. We also converted all the normalized feature values according to the Cohen’s *d* of the features between patients and controls, and thus, smaller values always indicate higher AD possibility. After that, we filtered out features with values 0, 1 or 0.5 for more than 95% patients. This is to discard features that are either not discriminative, or extremely dominated by patients or controls. After the filtering step, we have 112 cognitive features remained and used in experiments. Additional file 1: Table S1 presents these 112 cognitive features. We conducted the same process as above on the imaging features. Additional file 1: Table S2 presents these imaging features used in experiments.

#### Patient similarities from imaging features

Through the normalization and filtering steps as in “Data normalization” section, we have 86 normalized imaging features remained. We represent each patient using a vector of these features, denoted as ${\mathbf {r}}_p = [r_{p1}, r_{p2}, \cdots , r_{p86}]$, in which $r_{pi}$ ($i = 1, \cdots , 86$) is an imaging feature for patient *p*. We calculate the patient similarity from imaging features using the radial basis function (RBF) kernel, that is, $w_{pq} = \exp ( -\frac{\Vert {\mathbf {r}}_p - {\mathbf {r}}_q\Vert ^2}{2\sigma ^2})$, where $w_{pq}$ is the patient similarity used in $R_{\text {csim}}$.

## Results

### Baseline methods

We compare ${\mathtt {PLTR}}$ and ${\mathtt {PLTR_h}}$ with two baseline methods: the Bayesian Multi-Task Multi-Kernel Learning (${\mathtt {BMTMKL}}$) method [30] and the Kernelized Rank Learning (${\mathtt {KRL}}$) method [31].

#### Bayesian multi-task multi-kernel learning (${\mathtt {BMTMKL}}$)

${\mathtt {BMTMKL}}$ is a state-of-the-art baseline for biomarker prioritization. It was originally proposed to rank cell lines for drugs and won the DREAM 7 challenge [32]. In our study, ${\mathtt {BMTMKL}}$ uses the multi-task and multi-kernel learning within kernelized regression to predict cognitive feature values and learns parameters by conducting Bayesian inference. We use the patient similarity matrix calculated from FreeSurfer features as the kernels in ${\mathtt {BMTMKL}}$.

#### Kernelized rank learning (${\mathtt {KRL}}$)

KRL represents another state-of-the-art baseline for biomarker prioritization. In our study, ${\mathtt {KRL}}$ uses kernelized regression with a ranking loss to learn the ranking structure of patients and to predict the cognitive feature values. The objective of ${\mathtt {KRL}}$ is to maximize the hits among the top k of the ranking list. We use the patient similarity matrix calculated from FreeSurfer features as the kernels in ${\mathtt {KRL}}$.

**Fig. 1:**
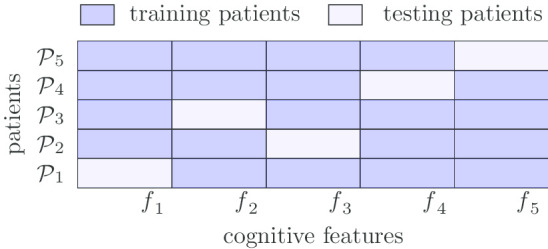
Data split for cross validation ($\texttt {CV}$)

**Fig. 2:**
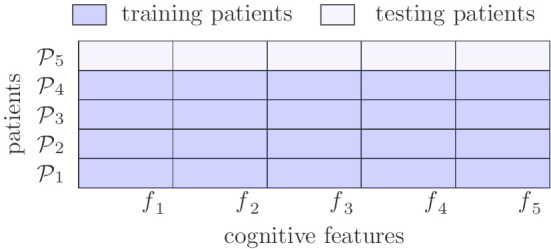
Data split for leave-out validation (${\texttt {LOV}}$)

### Training-testing data splits

Following the protocol in our preliminary study [8], we test our methods in two different settings: cross validation ($\texttt {CV}$) and leave-out validation (${\texttt {LOV}}$). In $\texttt {CV}$, we randomly split each patient’s cognitive tasks into 5 folds: all the features of a cognitive task will be either split into training or testing set. We use 4 folds for training and the rest fold for testing, and do such experiments 5 times, each with one of the 5 folds as the testing set. The overall performance of the methods is averaged over the 5 testing sets. This setting corresponds to the goal to prioritize additional cognitive tasks that a patient should complete. In ${\texttt {LOV}}$, we split patients (not patient tasks) into training and testing sets, and a certain patient and all his/her cognitive features will be either in the training set or in the testing set. This corresponds to the use scenario to identify the most relevant cognitive tasks that a new patient needs to take, based on the existing imaging information of the patient, when the patient has not completed any cognitive tasks. Figures 1 and 2 demonstrate the $\texttt {CV}$ and ${\texttt {LOV}}$ data split processes, respectively.

Please note that as presented in “Data normalization” section, for normalized cognitive features, smaller values always indicate more AD possibility. Thus, in both settings, we use the ranking list of normalized cognitive features of each patient as ground truth for training and testing.

### Parameters

We conduct grid search to identify the best parameters on each evaluation metric for each model. We use 0.3 and 0.1 as the value of $t_p$ and $t_o$, respectively. In the experimental results, we report the combinations of parameters that achieve the best performance on evaluation metrics. We implement ${\mathtt {PLTR}}$ and ${\mathtt {PLTR_h}}$ using Python 3.7.3 and Numpy 1.16.2, and run the experiments on Xeon E5-2680 v4 with 128G memory.

### Evaluation metrics

**Table 1:** Overall performance in $\texttt {CV}$

Method	Parameters		Feature level		Task level				
	d	$\lambda$	QH@5	WQH@5	NH$_1@1$	NH$_{2}@1$	NH$_{3}@1$	NH$_{5}@1$	NH$_{\text {all}}@1$
${\mathtt {PLTR}}$	10	–	$\underline{\textit{2.665}}\pm 0.07$	$3.136\pm 0.12$	$0.605\pm 0.03$	$0.701\pm 0.04$	$0.713\pm 0.05$	$0.725\pm 0.05$	$0.683\pm 0.04$
	10	–	$2.647\pm 0.08$	$\underline{\textit{3.191}}\pm 0.14$	$0.599\pm 0.03$	$0.677\pm 0.04$	$0.707\pm 0.04$	$0.725\pm 0.05$	$0.677\pm 0.04$
	10	–	$2.569\pm 0.08$	$2.957\pm 0.11$	$\textit{0.635}{\pm 0.03}$	$0.707\pm 0.04$	$0.689\pm 0.05$	$0.719\pm 0.04$	$0.653\pm 0.04$
	10	–	$2.623\pm 0.06$	$3.073\pm 0.09$	$0.623\pm 0.03$	$\underline{\textit{0.713}}\pm 0.05$	$0.707\pm 0.04$	$0.719\pm 0.04$	$0.671\pm 0.04$
	50	–	$2.467\pm 0.07$	$2.992\pm 0.11$	$0.605\pm 0.03$	$0.695\pm 0.04$	$\textit{0.725}\pm 0.06$	$0.725\pm 0.04$	$0.653\pm 0.04$
	30	–	$2.491\pm 0.07$	$3.080\pm 0.14$	$0.563\pm 0.04$	$0.689\pm 0.05$	$0.713\pm 0.04$	$\textit{0.749}\pm 0.04$	$\textit{0.689}\pm 0.03$
${\mathtt {PLTR_h}}$	10	–	*2.599* ± 0.09	3.111 ± 0.12	0.623 ± 0.02	0.671 ± 0.03	0.713 ± 0.03	0.719 ± 0.04	*0.707* ± 0.03
	10	–	$2.575\pm 0.08$	*3.115* ± 0.13	0.623 ± 0.03	0.677 ± 0.03	0.737 ± 0.04	0.749 ± 0.03	0.695 ± 0.03
	10	–	$2.419\pm 0.09$	$2.827\pm 0.12$	$\underline{\textit{0.647}}$ ± 0.03	0.695 ± 0.03	0.671 ± 0.03	0.707 ± 0.03	0.635 ± 0.03
	30	–	$2.138\pm 0.10$	$2.583\pm 0.18$	$0.629\pm 0.02$	$\textit{0.701}\pm 0.02$	0.695 ± 0.03	0.695 ± 0.04	0.593 ± 0.05
	50	–	$2.102\pm 0.07$	$2.470\pm 0.10$	$0.533\pm 0.03$	$0.677\pm 0.03$	$\underline{\textit{0.743}}$ ± 0.04	0.754 ± 0.03	0.629 ± 0.05
	30	–	$2.281\pm 0.07$	$2.768\pm 0.18$	$0.563\pm 0.03$	$0.689\pm 0.03$	0.707 ± 0.04	$\textit{0.760}$ ± 0.05	0.701 ± 0.05
${\mathtt {KRL}}$	–	2	*2.102* ± 0.26	*2.167* ± 0.37	*0.569* ± 0.03	*0.611* ± 0.05	*0.635* ± 0.04	*0.683* ± 0.03	0.689 ± 0.07
	–	1.5	2.078 ± 0.15	2.143 ± 0.25	0.503 ± 0.04	0.575 ± 0.05	0.617 ± 0.05	0.677 ± 0.04	$\underline{\textit{0.760}}$ ± 0.06
${\mathtt {BMTMKL}}$	–	–	*2.443* ± 0.12	*2.614* ± 0.20	*0.413* ± 0.07	*0.491* ± 0.08	*0.593* ± 0.05	$\underline{\textit{0.784}}$ ± 0.05	*0.749* ± 0.05

The column “*d*” corresponds to the latent dimension. The numbers in the form of $x\pm y$ represent the mean (*x*) and standard deviation (*y*). The best performance of each method is in italic. The best performance under each evaluation metric is underlined

#### Metrics on cognitive feature level

We use a metric named average feature hit at *k* (QH@*k*) as in our preliminary study [8] to evaluate the ranking performance,

10 $$\begin{aligned} {\text {QH}}@k({\tau }^q, \tilde{{\tau }}^q) = \sum \limits _{i = 1}^k {\mathbb {I}}(\tilde{{\tau }}^q_i \in {{\tau }^q(1:k)}), \end{aligned}$$

where ${\tau }^q$ is the ground-truth ranking list of all the features in all the tasks, ${\tau }^q(1:k)$ is the top *k* features in the list, $\tilde{{\tau }}^q$ is the predicted ranking list of all the features, and $\tilde{{\tau }}^q_i$ is the *i*th ranked features in $\tilde{{\tau }}^q$. That is, QH@*k* calculates the number of features among top *k* in the predicted feature lists that are also in the ground truth (i.e., hits). Higher QH@*k* values indicate better prioritization performance.

We use a second evaluation metric weighted average feature hit at *k* (WQH@*k*) as follows:

11 $$\begin{aligned} {\text {WQH}}@k({\tau }^q, \tilde{{\tau }}^q) = {\sum \limits _{j=1}^k QH@j({\tau }^q, \tilde{{\tau }}^q)}/k, \end{aligned}$$

that is, ${\text {WQH}}@k$ is a weighted version of ${\text {QH}}@k$ that calculates the average of ${\text {QH}}@j$ ($j = 1, \cdots , k$) over top *k*. Higher ${\text {WQH}}@k$ indicates more feature hits and those hits are ranked on top in the ranking list.

#### Metrics on cognitive task level

In in Peng et al. [8], we use the mean of the top-*g* normalized ground-truth scores/predicted scores on the features of each cognitive task for a patient as the score of that task for that patient. For each patient, we rank the tasks using their ground-truth scores and use the ranking as the ground-truth ranking of these tasks. Thus, these scores measure how much relevant to AD the task indicates for the patients. We use the predicted scores to rank cognitive tasks into the predicted ranking of the tasks. We define a third evaluation metric task hit at *k* (${\hbox {NH}}_g$@*k*) as follows to evaluate the ranking performance in terms of tasks,

12 $$\begin{aligned} {\text {NH}}_g@k({\tau }_g^n, \tilde{{\tau }}_g^n) = \sum \limits _{i = 1}^k {\mathbb {I}}(\tilde{{\tau }}^n_{gi} \in {{\tau }_g^n(1:k)}), \end{aligned}$$

where ${\tau }_g^n$/$\tilde{{\tau }}_g^n$ is the ground-truth/predicted ranking list of all the tasks using top-*g* question scores.

## Experimental results

### Overall Performance on $\texttt {CV}$

Table 1 presents the performance of ${\mathtt {PLTR}}$, ${\mathtt {PLTR_h}}$ and two baseline methods in the $\texttt {CV}$ setting. Note that overall, ${\mathtt {PLTR}}$ and ${\mathtt {PLTR_h}}$ have similar standard deviations; ${\mathtt {KRL}}$ and ${\mathtt {BMTMKL}}$ have higher standard deviations compared to ${\mathtt {PLTR}}$ and ${\mathtt {PLTR_h}}$. This indicates that ${\mathtt {PLTR}}$ and ${\mathtt {PLTR_h}}$ are more robust than ${\mathtt {KRL}}$ and ${\mathtt {BMTMKL}}$ for the prioritization tasks.

#### Comparison on cognitive feature level

For cognitive features from all tasks, ${\mathtt {PLTR}}$ is able to identify on average $2.665\pm 0.07$ out of the top-5 most relevant ground-truth cognitive features among its top-5 predictions (i.e., QH@5 = 2.665 ± 0.07). ${\mathtt {PLTR_h}}$ achieves similar performance as ${\mathtt {PLTR}}$, and identifies on average $2.599\pm 0.09$ most relevant ground-truth cognitive features on its top-5 predictions (i.e., QH@5 $=2.599\pm 0.09$). ${\mathtt {PLTR}}$ and ${\mathtt {PLTR_h}}$ significantly outperform the baseline methods in terms of all the evaluation metrics on cognitive feature level (i.e., QH@5 and WQH@5). Specifically, ${\mathtt {PLTR}}$ outperforms the best baseline method ${\mathtt {BMTMKL}}$ at $9.1\pm 3.7$% and $22.1 \pm 9.5$% on QH@5 and WQH@5, respectively. ${\mathtt {PLTR_h}}$ also outperforms ${\mathtt {BMTMKL}}$ at $6.4\pm 4.3$% and $19.2\pm 10.1$% on QH@5 and WQH@5, respectively. These experimental results demonstrate that among the top 5 features in the ranking list, ${\mathtt {PLTR}}$ and ${\mathtt {PLTR_h}}$ are able to rank more relevant features on top than the two state-of-the-art baseline methods and the positions of those hits are also higher than those in the baseline methods.

#### Comparison on cognitive task level

For the scenario to prioritize cognitive tasks that each patient should take, ${\mathtt {PLTR}}$ and ${\mathtt {PLTR_h}}$ are able to identify the top-1 most relevant task for $72.5\pm 6.0$% and $74.3\pm 4.0$% of all the patients when using 3 features to score cognitive tasks, respectively (i.e., ${\hbox {NH}}_3=0.725\pm 0.06$ for ${\mathtt {PLTR}}$ and ${\hbox {NH}}_3=0.743\pm 0.04$ for ${\mathtt {PLTR_h}}$). This indicates the strong power of ${\mathtt {PLTR}}$ and ${\mathtt {PLTR_h}}$ in prioritizing cognitive features and in recommending relevant cognition tasks for real clinical applications. We also find that ${\mathtt {PLTR}}$ and ${\mathtt {PLTR_h}}$ are able to outperform baseline methods on most of the metrics on cognitive task level (i.e., ${\hbox {NH}}_g@1$). ${\mathtt {PLTR}}$ outperforms the best baseline method at $11.6\pm 5.6$%, $16.7\pm 6.1$% and $14.2\pm 6.6$% on ${\hbox {NH}}_1@1$, ${\hbox {NH}}_2@1$ and ${\hbox {NH}}_3@1$, respectively. ${\mathtt {PLTR_h}}$ performs even better than ${\mathtt {PLTR}}$ on ${\hbox {NH}}_1@1$ and ${\hbox {NH}}_3@1$, in addition to that it outperforms the best performance of baseline methods at $13.7\pm 5.3$%, $14.7\pm 4.8$% and $17.0\pm 8.8$% on ${\hbox {NH}}_1@1$, ${\hbox {NH}}_2@1$ and ${\hbox {NH}}_3@1$, respectively. ${\mathtt {PLTR}}$ and ${\mathtt {PLTR_h}}$ perform slightly worse than baseline methods on ${\hbox {NH}}_5@1$ and ${\hbox {NH}}_{\text {all}}@1$ ($0.760\pm 0.05$ vs $0.784\pm 0.05$ on ${\hbox {NH}}_5@1$ and $0.707\pm 0.03$ vs $0.760\pm 0.06$ on ${\hbox {NH}}_{\text {all}}@1$). These experimental results indicate that ${\mathtt {PLTR}}$ and ${\mathtt {PLTR_h}}$ are able to push the most relevant task to the top of the ranking list than baseline methods when using a small number of features to score cognitive tasks. Note that in $\texttt {CV}$, each patient has only a few cognitive tasks in the testing set. Therefore, we only consider the evaluation at the top task in the predicted task rankings (i.e., only ${\hbox {NH}}_g@1$ in Table 1).

Table 1 also shows that ${\mathtt {PLTR_h}}$ outperforms ${\mathtt {PLTR}}$ on most of the metrics on cognitive task level (i.e., ${\hbox {NH}}_g@1$). ${\mathtt {PLTR_h}}$ outperforms ${\mathtt {PLTR}}$ at 1.9 ± 0.5%, 2.5 ± 1.2%, 1.5 ± 0.3% and 2.6 ± 0.9% on ${\hbox {NH}}_1$@1, ${\hbox {NH}}_3$@1, ${\hbox {NH}}_5$@1 and ${\hbox {NH}}_\text {all}$@1, respectively. This indicates that generally ${\mathtt {PLTR_h}}$ is better than ${\mathtt {PLTR}}$ on ranking cognitive tasks in $\texttt {CV}$ setting. The reason could be that the hinge-based loss functions with pre-defined margins can enable significant difference between the scores of relevant features and irrelevant features, and thus effectively push relevant features upon irrelevant features.

### Overall performance on ${\texttt {LOV}}$

**Table 2:** Overall performance in ${\texttt {LOV}}$ on 26 testing patients

Method	Feature level		Task level									
	QH@5	WQH@5	NH$_1$@1	NH$_1$@5	NH$_2$@1	NH$_2$@5	NH$_3$@1	NH$_3$@5	NH$_5$@1	NH$_5$@5	NH$_{\text {all}}$@1	NH$_{\text {all}}$@5
${\mathtt {PLTR}}$	$\underline{\textit{1.615}}$	$\underline{\textit{1.906}}$	0.846	3.231	0.577	3.385	0.231	3.654	0.308	3.346	0.808	3.692
	1.500	1.778	$\underline{\textit{0.846}}$	*3.269*	$\underline{\textit{0.577}}$	$\underline{\textit{3.538}}$	0.269	3.654	0.269	3.269	0.808	3.577
	1.538	1.856	0.846	3.192	0.577	3.423	*0.308*	$\underline{\textit{3.731}}$	0.346	3.346	0.808	3.615
	1.577	1.851	0.846	3.192	0.577	3.462	0.308	3.654	$\underline{\textit{0.346}}$	*3.462*	0.808	3.654
	1.615	1.906	0.846	3.231	0.577	3.385	0.231	3.654	0.308	3.346	$\underline{\textit{0.808}}$	*3.692*
${\mathtt {PLTR_h}}$	$\underline{\textit{1.615}}$	1.836	$\underline{\textit{0.846}}$	3.192	$\underline{\textit{0.577}}$	*3.500*	*0.269*	3.731	$\underline{\textit{0.346}}$	3.731	$\underline{\textit{0.808}}$	4.154
	1.538	*1.891*	0.846	3.192	0.577	3.500	0.269	3.731	0.346	3.615	0.808	4.038
	1.538	1.856	0.769	*3.308*	0.577	3.462	0.269	3.615	0.308	3.385	0.808	3.500
	1.538	1.712	0.846	3.115	0.577	3.423	0.154	$\underline{\textit{3.731}}$	0.308	*3.808*	0.808	$\underline{\textit{4.269}}$
${\mathtt {KRL}}$	*1.423*	1.656	0.615	2.615	$\underline{\textit{0.577}}$	3.308	0.038	3.577	$\underline{\textit{0.346}}$	$\underline{\textit{3.962}}$	$\underline{\textit{0.808}}$	$\underline{\textit{4.269}}$
	1.346	*1.881*	0.577	2.615	0.577	3.308	0.038	3.577	0.346	3.962	0.808	4.269
	1.346	1.435	*0.808*	$\underline{\textit{3.423}}$	0.538	*3.500*	$\underline{\textit{0.346}}$	$\underline{\textit{3.731}}$	0.154	3.423	0.808	3.538
${\mathtt {BMTMKL}}$	*0.423*	*0.212*	$\underline{\textit{0.846}}$	*2.615*	$\underline{\textit{0.577}}$	*3.308*	*0.038*	*3.577*	$\underline{\textit{0.346}}$	*3.769*	$\underline{\textit{0.808}}$	$\underline{\textit{4.269}}$

The column “n” corresponds to the number of hold-out testing patients. The bset performance of each method is in italic.The best performance under each evaluation metric is *underlined*

**Table 3:** Overall Performance in ${\texttt {LOV}}$ on 52 testing patients

Method	Feature level		Task level									
	QH@5	WQH@5	NH$_1$@1	NH$_1$@5	NH$_2$@1	NH$_2$@5	NH$_3$@1	NH$_3$@5	NH$_5$@1	NH$_5$@5	NH$_{\text {all}}$@1	NH$_{\text {all}}$@5
${\mathtt {PLTR}}$	*1.385*	*1.668*	0.788	3.212	0.423	3.654	0.115	3.750	0.288	3.423	0.788	3.423
	1.327	1.616	$\underline{\textit{0.808}}$	$\underline{\textit{3.269}}$	0.423	3.654	0.115	3.731	0.173	3.423	0.788	3.404
	1.327	1.652	0.788	3.212	*0.423*	$\underline{\textit{3.712}}$	*0.115*	$\underline{\textit{3.750}}$	0.269	3.423	0.788	3.404
	1.308	1.616	0.788	3.154	0.423	3.654	0.115	3.712	*0.288*	3.481	0.788	3.615
	1.288	1.581	0.808	3.173	0.423	3.596	0.115	3.750	0.192	*3.519*	0.788	3.635
	1.269	1.616	0.808	3.115	0.423	3.635	0.115	3.731	0.250	3.481	$\underline{\textit{0.788}}$	*3.635*
${\mathtt {PLTR_h}}$	$\underline{\textit{1.404}}$	1.656	0.750	2.827	*0.404*	3.250	0.173	3.481	$\underline{\textit{0.385}}$	3.596	$\underline{\textit{0.788}}$	$\underline{\textit{4.154}}$
	1.365	$\underline{\textit{1.695}}$	0.731	2.808	0.365	3.308	0.173	3.462	0.365	3.596	0.788	4.154
	1.327	1.562	$\underline{\textit{0.808}}$	3.077	0.404	3.365	0.135	3.577	0.250	3.673	0.788	4.115
	1.327	1.605	0.769	*3.154*	0.385	*3.596*	0.135	3.712	0.212	3.519	0.788	3.577
	1.308	1.609	0.769	2.904	0.385	3.308	$\underline{\textit{0.192}}$	3.442	0.365	3.654	0.788	4.154
	1.327	1.605	0.769	3.154	0.385	3.596	0.135	*3.712*	0.212	3.519	0.788	3.577
	1.288	1.545	0.788	3.000	0.404	3.385	0.154	3.558	0.308	*3.712*	0.788	4.154
${\mathtt {KRL}}$	*1.173*	*1.548*	0.096	2.577	0.385	*3.231*	0.077	*3.385*	*0.346*	$\underline{\textit{3.808}}$	$\underline{\textit{0.788}}$	$\underline{\textit{4.154}}$
	1.173	1.534	*0.154*	*2.615*	0.250	3.192	0.077	3.385	0.346	3.712	0.788	4.154
	1.096	1.437	0.077	2.577	*0.462*	3.231	0.077	3.385	0.346	3.808	0.788	4.154
	0.423	0.504	0.019	2.019	0.038	2.500	*0.115*	2.481	0.115	2.712	0.019	2.673
${\mathtt {BMTMKL}}$	*0.403*	*0.255*	$\underline{\textit{0.808}}$	*2.577*	$\underline{\textit{0.481}}$	*3.231*	*0.077*	*3.385*	*0.346*	*3.596*	$\underline{\textit{0.788}}$	$\underline{\textit{4.154}}$

The column “n” corresponds to the number of hold-out testing patients. The best performance of each model is in italic. The best performance under each evaluation metric is upon underline.

Tables 2 and 3 present the performance of ${\mathtt {PLTR}}$, ${\mathtt {PLTR_h}}$ and two baseline methods in the ${\texttt {LOV}}$ setting. Due to space limit, we did not present the standard deviations in the tables, but they have similar trends as those in Table 1. We first hold out 26 (Table 2) and 52 (Table 3) AD patients as testing patients, respectively. We determine these hold-out AD patients as the ones that have more than 10 similar AD patients in the training set with corresponding patient similarities higher than 0.67 and 0.62, respectively.

#### Comparison on cognitive feature level

Tables 2 and 3 show that ${\mathtt {PLTR}}$ and ${\mathtt {PLTR_h}}$ significantly outperform the baseline methods in terms of all the evaluation metrics on cognitive feature level (i.e., QH@5 and WQH@5), which is consistent with the experimental results in $\texttt {CV}$ setting. When 26 patients are hold out for testing, with parameters $\alpha = 0.5$, $\beta = 1.5$, $\gamma = 1.0$ and $\text {d} = 30$, ${\mathtt {PLTR}}$ outperforms the best baseline method ${\mathtt {KRL}}$ at 13.4% and 1.3% on QH@5 and WQH@5, respectively. The performance of ${\mathtt {PLTR_h}}$ is very comparable with that of ${\mathtt {PLTR}}$ ” ${\mathtt {PLTR_h}}$ outperforms ${\mathtt {KRL}}$ at 13.4% and 0.5% on QH@5 and WQH@5, respectively. When 52 patients are hold out for testing, with parameters $\alpha = 0.5$, $\beta = 0.5$, $\gamma = 1.0$ and $\text {d} = 50$, ${\mathtt {PLTR}}$ outperforms the best baseline method ${\mathtt {KRL}}$ at 18.1% and 7.8% on QH@5 and WQH@5, respectively. ${\mathtt {PLTR_h}}$ even performs better than ${\mathtt {PLTR}}$ in this setting. In addition, ${\mathtt {PLTR_h}}$ outperforms ${\mathtt {KRL}}$ at 19.7% and 9.5% on QH@5 and WQH@5, respectively. These experimental results demonstrate that for new patients, ${\mathtt {PLTR}}$ and ${\mathtt {PLTR_h}}$ are able to rank more relevant features to the top of the ranking list than the two baseline methods. They also indicate that for new patients, ranking based methods (e.g., ${\mathtt {PLTR}}$ and ${\mathtt {PLTR_h}}$) are more effective than regression based methods (e.g., ${\mathtt {KRL}}$ and ${\mathtt {BMTMKL}}$) for biomarker prioritization.

#### Comparison on cognitive task level

Table 2 also shows that when 26 patients are hold out for testing, ${\mathtt {PLTR}}$ and ${\mathtt {PLTR_h}}$ are both able to identify the top most relevant questionnaire for 84.6% of the testing patients (i.e., 22 patients) under ${\hbox {NH}}_1@1$. Table 3 shows that when 52 patients are hold out for testing, ${\mathtt {PLTR}}$ and ${\mathtt {PLTR_h}}$ are both able to identify for 80.8% of the testing patients (i.e., 42 patients) under ${\hbox {NH}}_1@1$. Note that the hold-out testing patients in ${\texttt {LOV}}$ do not have any cognitive features. Therefore, the performance of ${\mathtt {PLTR}}$ and ${\mathtt {PLTR_h}}$ as above demonstrates their strong capability in identifying most AD related cognitive features based on imaging features only. We also find that ${\mathtt {PLTR}}$ and ${\mathtt {PLTR_h}}$ are able to achieve similar or even better results compared to baseline methods in terms of the evaluation metrics on cognitive task level (i.e., ${\hbox {NH}}_g$@1 and ${\hbox {NH}}_g$@5). When 26 patients are hold out for testing, ${\mathtt {PLTR}}$ and ${\mathtt {PLTR_h}}$ outperform the baseline methods in terms of ${\hbox {NH}}_g$@1 (i.e., $g = 1, 2 \ldots 5$). They are only slightly worse than ${\mathtt {KRL}}$ on ranking relevant tasks on their top-5 of predictions when $g = 1$ or $g = 5$ (3.308 vs 3.423 on ${\hbox {NH}}_1$@5 and 3.808 vs 3.962 on ${\hbox {NH}}_5$@5). When 52 patients are hold out for testing, ${\mathtt {PLTR}}$ and ${\mathtt {PLTR_h}}$ also achieve the best performance on most of the evaluation metrics. They are only slightly worse than ${\mathtt {KRL}}$ on ${\hbox {NH}}_2$@1, ${\hbox {NH}}_5$@5 (0.423 vs 0.481 on ${\hbox {NH}}_2$@1 and 3.712 vs 3.808 on ${\hbox {NH}}_5$@5). These experimental results demonstrate that among top 5 tasks in the ranking list, ${\mathtt {PLTR}}$ and ${\mathtt {PLTR_h}}$ rank more relevant task on top than ${\mathtt {KRL}}$.

It’s notable that in Tables 2 and 3, as the number of features used to score cognitive tasks (i.e., *g* in ${\hbox {NH}}_g@k$) increases, the performance of all the methods in ${\hbox {NH}}_g@1$ first declines and then increases. This may indicate that as *g* increases, irrelevant features which happen to have relatively high scores will be included in scoring tasks, and thus degrade the model performance on ${\hbox {NH}}_g@1$. However, generally, the scores of irrelevant features are considerably lower than those of relevant ones. Thus, as more features are included, the scores for tasks are more dominated by the scores of relevant features and thus the performance increases.

We also find that ${\mathtt {BMTMKL}}$ performs poorly on ${\hbox {NH}}_3@1$ in both Tables 2 and 3. This indicates that ${\mathtt {BMTMKL}}$, a regression-based method, could not well rank relevant features and irrelevant features. It’s also notable that generally the best performance for the 26 testing patients is better than that for 52 testing patients. This may be due to that the similarities between the 26 testing patients and their top 10 similar training patients are higher than those for the 52 testing patients. The high similarities enable accurate latent vectors for testing patients.

Tables 2 and 3 also show that ${\mathtt {PLTR_h}}$ is better than ${\mathtt {PLTR}}$ on ranking cognitive tasks in ${\texttt {LOV}}$ setting. When 26 patients are hold out for testing, ${\mathtt {PLTR_h}}$ outperforms ${\mathtt {PLTR}}$ on ${\hbox {NH}}_1$@5, ${\hbox {NH}}_5$@5 and ${\hbox {NH}}_\text {all}$@5 and achieves very comparable performance on the rest metrics. When 52 patients are hold out for testing, ${\mathtt {PLTR_h}}$ is able to achieve better performance than ${\mathtt {PLTR}}$ on QH@5, WQH@5, ${\hbox {NH}}_3$@1, ${\hbox {NH}}_5$@1, ${\hbox {NH}}_5$@5 and ${\hbox {NH}}_\text {all}$@5 and also achieves very comparable performance on the rest metrics. Generally, ${\mathtt {PLTR_h}}$ outperforms ${\mathtt {PLTR}}$ in terms of metrics on cognitive task level. This demonstrates the effectiveness of hinge loss-based methods in separating relevant and irrelevant features during modeling.

## Discussion

Our experimental results show that when ${\hbox {NH}}_1@1$ achieves its best performance of 0.846 for the 26 testing patients in the ${\texttt {LOV}}$ setting (i.e., the first row block in Table 2), the task that is most commonly prioritized for the testing patients is Rey Auditory Verbal Learning Test (RAVLT), including the following cognitive features: (1) trial 1 total number of words recalled; (2) trial 2 total number of words recalled; (3) trial 3 total number of words recalled; (4) trial 4 total number of words recalled; (5) trial 5 total number of words recalled; (6) total Score; (7) trial 6 total number of words recalled; (8) list B total number of words recalled; (9) 30 min delay total; and (10) 30 min delay recognition score. RAVLT is also the most relevant task in the ground truth if tasks are scored correspondingly. RAVLT assesses learning and memory, and has shown promising performance in early detection of AD [33]. A number of studies have reported high correlations between various RAVLT scores with different brain regions [34]. For instance, RAVLT recall is associated with medial prefrontal cortex and hippocampus; RAVLT recognition is highly correlated with thalamic and caudate nuclei. In addition, genetic analysis of *APOE* $\varepsilon$4 allele, the most common variant of AD, reported its association with RAVLT score in an early-MCI (EMCI) study [26]. The fact that RAVLT is prioritized demonstrates that ${\mathtt {PLTR}}$ is powerful in prioritizing cognitive features to assist AD diagnosis.

Similarly, we find the top-5 most frequent cognitive tasks corresponding to the performance at ${\hbox {NH}}_3@5$ = 3.731 for the 26 hold-out testing patients. They are: Functional Assessment Questionnaire (FAQ), Clock Drawing Test (CDT), Weschler’s Logical Memory Scale (LOGMEM), Rey Auditory Verbal Learning Test (RAVLT), and Neuropsychiatric Inventory Questionnaire (NPIQ). In addition to RAVLT discussed above, other top prioritized cognitive tasks have also been reported to be associated with AD or its progression. In an MCI to AD conversion study, FAQ, NPIQ and RAVLT showed significant difference between MCI-converter and MCI-stable groups [35]. We also notice that for some testing subjects, ${\mathtt {PLTR}}$ is able to very well reconstruct their ranking structures. For example, when ${\hbox {NH}}_3@5$ achieves its optimal performance 3.731, for a certain testing subject, her top-5 predicted cognitive tasks RAVLT, LOGMEM, FAQ, NPIQ and CDT are exactly the top-5 cognitive tasks in the ground truth. These evidences further demonstrate the diagnostic power of our method.

## Conclusions

We have proposed a novel machine learning paradigm to prioritize cognitive assessments based on their relevance to AD at the individual patient level. The paradigm tailors the cognitive biomarker discovery and cognitive assessment selection process to the brain morphometric characteristics of each individual patient. It has been implemented using newly developed learning-to-rank method ${\mathtt {PLTR}}$ and ${\mathtt {PLTR_h}}$. Our empirical study on the ADNI data has produced promising results to identify and prioritize individual-specific cognitive biomarkers as well as cognitive assessment tasks based on the individual’s structural MRI data. In addition, ${\mathtt {PLTR_h}}$ shows better performance than ${\mathtt {PLTR}}$ on ranking cognitive assessment tasks. The resulting top ranked cognitive biomarkers and assessment tasks have the potential to aid personalized diagnosis and disease subtyping, and to make progress towards enabling precision medicine in AD.

## Supplementary information

**Additional file 1.** Supplementary materials.

## Data Availability

The dataset supporting the conclusions of this article is available in the Alzheimer’s Disease Neuroimaging Initiative (ADNI) [25].

## Abbreviations

AD: Alzheimer’s Disease
MRI: Magnetic resonance imaging
ADNI: Alzheimer’s Disease Neuroimaging Initiative
LETOR: Learning-to-Rank
PET: Positron emission tomography
MCI: Mild Cognitive Impairment
HC: Healthy control
ADAS: Alzheimer’s Disease Assessment Scale
CDR: Clinical Dementia Rating Scale
FAQ: Functional Assessment Questionnaire
GDS: Geriatric Depression Scale
MMSE: Mini-Mental State Exam
MODHACH: Modified Hachinski Scale
NPIQ: Neuropsychiatric Inventory Questionnaire
BNT: Boston Naming Test
CDT: Clock Drawing Test
DSPAN: Digit Span Test
DSYM: Digit Symbol Test
FLUENCY: Category Fluency Test
LOGMEM: Weschler’s Logical Memory Scale
RAVLT: Rey Auditory Verbal Learning Test
TRAIL: Trail Making Test
RBF: Radial Basis Function
PLTR: Joint Push and Learning-to-Rank Method
${\hbox {PLTR}}_\mathbf{h }$: Joint Push and Learning-to-Rank Method using Hinge Loss
BMTMKL: Bayesian Multi-Task Multi-Kernel Learning
KRL: Kernelized Rank Learning
CV: Cross Validation
LOV: Leave-Out Validation
QH@*k*: Average Feature Hit at *k*
WQH@*k*: Weighted Average Feature Hit at *k*
${\text {NH}}_g$@*k*: Task Hit at *k*
APOE: Apolipoprotein E
EMCI: Early-MCI
