# Supplementary material for: Cognitive biomarker prioritization in Alzheimer’s Disease using brain morphometric data
Source: BMC Med Inform Decis Mak. 2020 Dec 2;20:319. doi: 10.1186/s12911-020-01339-z (PMC7709267; doi:10.1186/s12911-020-01339-z)
Supplement: Supplementary file 1 — Additional file 1. Supplementary materials. [file 12911_2020_1339_MOESM1_ESM.pdf]

RESEARCH

# Cognitive Biomarker Prioritization in Alzheimer's Disease using Brain Morphometric Data (Supplementary Materials)

Bo Peng[1], Xiaohui Yao[2], Shannon L. Risacher[3], Andrew J. Saykin[3], Li Shen[2], Xia Ning[1*] and for the ADNI[†]

## 1 Features used in experiments

Table S1 presents the cognitive features that are used in our experiments after data processing. Table S2 presents the image features that are used in our experiments after data processing.

Table S1: Cognitive Features Used in Experiments

| Task | Measurement/Question |
|---|---|
| ADAS | Q1 - Word Recall |
| ADAS | Q2 - Commands |
| ADAS | Q3 - Construction |
| ADAS | Q4 - Delayed Word Recall |
| ADAS | Q5 - Naming |
| ADAS | Q6 - Ideational Praxis |
| ADAS | Q7 - Orientation |
| ADAS | Q8 - Word Recognition |
| ADAS | Q9 - Recall Instructions |
| ADAS | Q10 - Spoken Language |
| ADAS | Q11 - Word Finding |
| ADAS | Q12 - Comprehension |
| ADAS | Q14 - Number Cancellation |
| ADAS | Sub-Total (classic 70 point total that excludes Q4 (Delayed Word Recall) and Q14 (Number Cancellation)) |
| ADAS | Total (85 point total that includes Q4 (Delayed Word Recall) and Q14 (Number Cancellation)) |
| CDR | CDR Memory score |
| CDR | CDR Orientation score |
| CDR | CDR Judgement and Problem Solving score |
| CDR | CDR Community Affairs score |
| CDR | CDR Home & Hobbies score |
| CDR | CDR Personal Care score |
| CDR | Global CDR score |
| CDR | CDR Sum-of-Boxes score |
| FAQ | Any difficulty in writing checks paying bills and/or balancing checkbook? |
| FAQ | Any difficulty in assembling tax records business affairs or other papers? |
| FAQ | Any difficulty in shopping alone for clothes household necessities or groceries? |
| FAQ | Any difficulty in playing a game of skill such as bridge or chess or working on a hobby? |
| FAQ | Any difficulty in heating water making a cup of coffee or turing off the stove? |
| FAQ | Any difficulty in preparing a balanced meal? |
| FAQ | Any difficulty in keeping track of current events? |
| FAQ | Any difficulty in paying attention to and understanding a TV program book or magazine? |

Continued on next page

**Table S1 – continued from previous page**

| Task | Measurement/Question |
| --- | --- |
| FAQ | Any difficulty in remembering appointments family occasions holidays medications? |
| FAQ | Any difficulty in traveling out of the neighborhood driving or arranging to take public transportation? |
| FAQ | Total FAQ score |
| GDS | Q2 - Have you dropped many of your activities and interests? (YES = 1 point) |
| GDS | Q4 - Do you often get bored? (YES = 1 point) |
| GDS | Q8 - Do you often feel helpless? (YES = 1 point) |
| GDS | Q10 - Do you feel you have more problems with memory than most? (YES = 1 point) |
| GDS | Q15 - Do you think that most people are better off than you are? (YES = 1 point) |
| GDS | GDS Total Score |
| MMSE | Q1 - What is today's date? |
| MMSE | Q2 - What is the year? |
| MMSE | Q3 - What is the month? |
| MMSE | Q4 - What day of the week is today? |
| MMSE | Q5 - What season is it? |
| MMSE | Q6 - What is the name of this hospital (clinic place)? |
| MMSE | Q7 - What floor are we on? |
| MMSE | Q8 - What town or city are we in? |
| MMSE | Q9 - What county (district borough area) are we in? |
| MMSE | Q15 - L |
| MMSE | Q16 - R |
| MMSE | Q17 - O |
| MMSE | Q18 - W |
| MMSE | Q19 - Ball |
| MMSE | Q20 - Flag |
| MMSE | Q21 - Tree |
| MMSE | Q24 - Say: Repeat after me: no ifs ands or buts. |
| MMSE | Q30 - Present the participant with the Construction Stimulus page. Say: Copy this design. |
| MMSE | MMSE Total Score |
| NIP | QA - Does participant believe that others are stealing from him/her or planning to harm him/her in some way? (Delusions) |
| NIP | QC - Is the participant stubborn and resistive to help from others? (Agitation/Aggression) |

Continued on next page

**Table S1 – continued from previous page**

| Task | Measurement/Question |
|---|---|
| NIP | QD - Does the participant act as if he/she is sad or in low spirits? Does he/she cry? (Depression/Dysphoria) |
| NIP | QE - Does the participant become upset when separated from you? Does he/she have any other signs of nervousness such as shortness of breath sighing being unable to relax or feeling excessively tense? (Anxiety) |
| NIP | QG - Does the participant seem less interested in his/her usual activities and in the activities and plans of others? (Apathy/Indifference) |
| NIP | QH - Does the participant seem to act impulsively? For example does the participant talk to strangers as if he/she knows them or does the participant say things that may hurt people's feelings? (Disinhibition) |
| NIP | QI - Is the participant impatient or cranky? Does he/she have difficulty coping with delays or waiting for planned activities? (Irritability/Lability) |
| NIP | QJ - Does the participant engage in repetitive activities such as pacing around the house handling buttons wrapping strings or doing other things repeatedly? (Abberant Motor Behavior) |
| NIP | QK - Does the participant awaken you during the night rise too early in the morning or take excessive naps during the day? (Sleep) |
| NIP | QL - Has the participant lost or gained weight or had a change in the food he/she likes? (Appetite) |
| NIP | Total NPIQ score |
| BNT | Baseline total ANART score (Total number of errors on the ANART) |
| BNT | Boston Naming Test - Number of spontaneously given correct responses |
| BNT | Boston Naming Test - Number of semantic cues given |
| BNT | Boston Naming Test - Number of phonemic cues given |
| BNT | Boston Naming Test - Number of correct responses following a phonemic cue |
| BNT | Boston Naming Test Total Score |
| CLOCK | Clock Drawing - Symmetry of number placement |
| CLOCK | Clock Drawing - Correctness of numbers |
| CLOCK | Clock Drawing - Presence of the two hands |
| CLOCK | Clock Drawing - Presence of the two hands set to ten after eleven |
| CLOCK | Clock Drawing Total Score |
| CLOCK | Clock Copy - Symmetry of number placement |
| CLOCK | Clock Copy - Correctness of numbers |
| CLOCK | Clock Copy - Presence of the two hands set to ten after eleven |
| CLOCK | Clock Copy Total Score |
| DIGITSPAN | Digit Span Forward - Total Correct |

Continued on next page

**Table S1 – continued from previous page**

| Task | Measurement/Question |
|---|---|
| DIGITSPAN | Digit Span Forward - Length |
| DIGITSPAN | Digit Span Backward - Total Correct |
| DIGITSPAN | Digit Span Backward - Length |
| DIGITSYM | Digit Symbol Total Score |
| FLUENCY | Category Fluency (Animals) - Total Correct |
| FLUENCY | Category Fluency (Animals) - Perseverations |
| FLUENCY | Category Fluency (Vegetables) - Total Correct |
| FLUENCY | Category Fluency (Vegetables) - Perseverations |
| FLUENCY | Category Fluency (Vegetables) - Intrusions |
| LOGMEM | Wechler's Logical Memory Immediate Recall - Total Number of Story Units Recalled |
| LOGMEM | Wechler's Logical Memory Delayed Recall - Total Number of Story Units Recalled |
| RAVLT | Trial 1 Total Number of Words Recalled |
| RAVLT | Trial 2 Total Number of Words Recalled |
| RAVLT | Trial 3 Total Number of Words Recalled |
| RAVLT | Trial 4 Total Number of Words Recalled |
| RAVLT | Trial 5 Total Number of Words Recalled |
| RAVLT | RAVLT Total Score |
| RAVLT | Trial 6 Total Number of Words Recalled |
| RAVLT | List B Total Number of Words Recalled |
| RAVLT | 30 Minute Delay Total |
| RAVLT | 30 Minute Delay Recognition Score |
| TRAIL | Trails A - Time to Complete (seconds) |
| TRAIL | Trails A - Errors of Omission |
| TRAIL | Trails B - Time to complete (seconds) |
| TRAIL | Trails B - Errors of Omission |
| TRAIL | Trails B - Errors of Commission |

The "Task" column corresponds to the task that the cognitive feature belongs to. The "Measurement/Question" column corresponds to the measurement/question that the cognitive is.

Table S2: Baseline Imaging Features Used in Experiments.

| Feature | Description |
| --- | --- |
| LCerebWM | Left Cerebral White Matter Volume |
| LCerebCtx | Left Cerebral Cortex Volume |
| RCerebCtx | Right Cerebral Cortex Volume |
| LLatVent | Left Lateral Ventricle Volume |
| RLatVent | Right Lateral Ventricle Volume |
| LInfLatVent | Left Inferior Lateral Ventricle Volume |
| RInfLatVent | Right Inferior Lateral Ventricle Volume |
| LPutamVol | Left Putamen Volume |
| RPutamVol | Right Putamen Volume |
| LHippVol | Left Hippocampus Volume |
| RHippVol | Right Hippocampus Volume |
| LAmygVol | Left Amygdala Volume |
| RAmygVol | Right Amygdala Volume |
| LAccumVol | Left Accumbens Volume |
| RAccumVol | Right Accumbens Volume |
| CSF | CSF Volume |
| CC_Post | Posterior Corpus Collosum Volume |
| CC_MidPost | Mid-Posterior Corpus Collosum Volume |
| CC_Cent | Central Corpus Collosum Volume |
| CC_MidAnt | Mid-Anterior Corpus Collosum Volume |
| CC_Ant | Anterior Corpus Collosum Volume |
| LBanksSTS | Left Banks Of The Superior Temporal Sulcus Thickness |
| RBanksSTS | Right Banks Of The Superior Temporal Sulcus Thickness |
| LCaudAntCing | Left Caudal Anterior Cingulate Thickness |
| RCaudAntCing | Right Caudal Anterior Cingulate Thickness |
| LCaudMidFrontal | Left Caudal Middle Frontal Gyri Thickness |
| RCaudMidFrontal | Right Caudal Middle Frontal Gyri Thickness |
| RCuneus | Right Cuneus Thickness |
| LEntCtx | Left Entorhinal Cortex Thickness |
| REntCtx | Right Entorhinal Cortex Thickness |
| LFusiform | Left Fusiform Gyri Thickness |
| RFusiform | Right Fusiform Gyri Thickness |
| LInfParietal | Left Inferior Parietal Gyri Thickness |
| RInfParietal | Right Inferior Parietal Gyri Thickness |
| LInfTemporal | Left Inferior Temporal Gyri Thickness |

Continued on next page

**Table S2 – continued from previous page**

| Feature | Description |
|---|---|
| RInfTemporal | Right Inferior Temporal Gyri Thickness |
| LIsthmCing | Left Isthmus Cingulate Thickness |
| RIsthmCing | Right Isthmus Cingulate Thickness |
| LLatOccipital | Left Lateral Occipital Gyri Thickness |
| RLatOccipital | Right Lateral Occipital Gyri Thickness |
| LLatOrbFrontal | Left Lateral Orbitofrontal Gyri Thickness |
| RLatOrbFrontal | Right Lateral Orbitofrontal Gyri Thickness |
| LLingual | Left Lingual Gyri Thickness |
| RLingual | Right Lingual Gyri Thickness |
| LMedOrbFrontal | Left Medial Orbitofrontal Thickness |
| RMedOrbFrontal | Right Medial Orbitofrontal Thickness |
| LMidTemporal | Left Middle Temporal Gyri Thickness |
| RMidTemporal | Right Middle Temporal Gyri Thickness |
| LParahipp | Left Paracentral Lobule Thickness |
| RParahipp | Right Paracentral Lobule Thickness |
| LParacentral | Left Parahippocampal Gyri Thickness |
| RParacentral | Right Parahippocampal Gyri Thickness |
| LParsOper | Left Pars Opercularis Thickness |
| RParsOper | Right Pars Opercularis Thickness |
| LParsOrb | Left Pars Orbitalis Thickness |
| RParsOrb | Right Pars Orbitalis Thickness |
| LParsTriang | Left Pars Triangularis Thickness |
| RParsTriang | Right Pars Triangularis Thickness |
| LPostCent | Left Postcentral Gyri Thickness |
| RPostCent | Right Postcentral Gyri Thickness |
| LPostCing | Left Posterior Cingulate Thickness |
| RPostCing | Right Posterior Cingulate Thickness |
| LPrecent | Left Precentral Gyri Thickness |
| RPrecent | Right Precentral Gyri Thickness |
| LPrecuneus | Left Precuneus Thickness |
| RPrecuneus | Right Precuneus Thickness |
| LRostAntCing | Left Rostral Anterior Cingulate Thickness |
| RRostAntCing | Right Rostral Anterior Cingulate Thickness |
| LRostMidFrontal | Left Rostral Middle Frontal Gyri Thickness |
| RRostMidFrontal | Right Rostral Middle Frontal Gyri Thickness |
| LSupFrontal | Left Superior Frontal Gyri Thickness |

Continued on next page

**Table S2 – continued from previous page**

| Feature | Description |
|---|---|
| RSupFrontal | Right Superior Frontal Gyri Thickness |
| LSupParietal | Left Superior Parietal Gyri Thickness |
| RSupParietal | Right Superior Parietal Gyri Thickness |
| LSupTemporal | Left Superior Temporal Gyri Thickness |
| RSupTemporal | Right Superior Temporal Gyri Thickness |
| LSupramarg | Left Supramarginal Gyri Thickness |
| RSupramarg | Right Supramarginal Gyri Thickness |
| LFrontalPole | Left Frontal Pole Thickness |
| RFrontalPole | Right Frontal Pole Thickness |
| LTemporalPole | Left Temporal Pole Thickness |
| RTemporalPole | Right Temporal Pole Thickness |
| LTransvTemporal | Left Transverse Temporal Pole Thickness |
| RTransvTemporal | Right Transverse Temporal Pole Thickness |
| LHippGM | Left Hippocampus Gray Matter Volume |
| RHippGM | Right Hippocampus Gray Matter Volume |

The "Feature" column corresponds to the feature name. The "Description" column corresponds to the description of the feature.

**Author details**

[1]The Ohio State University, Columbus, US. [2]University of Pennsylvania, Philadelphia, US. [3]Indiana University, Indianapolis, US.

**References**

*Correspondence: ning.104@osu.edu

[1]The Ohio State University, Columbus, US

Full list of author information is available at the end of the article

†Data used in preparation of this article were obtained from the Alzheimer's Disease Neuroimaging Initiative (ADNI) database (adni.loni.usc.edu). As such, the investigators within the ADNI contributed to the design and implementation of ADNI and/or provided data but did not participate in analysis or writing of this report. A complete listing of ADNI investigators can be found at: https://adni.loni.usc.edu/wp-content/uploads/how_to_apply/ADNI_Data_Use_Agreement.pdf.
